# Speciation and Bio-Imaging of Chromium in *Taraxacum officinale* Using HPLC Post-column ID-ICP-MS, High Resolution MS and Laser Ablation ICP-MS Techniques

**DOI:** 10.3389/fchem.2022.863387

**Published:** 2022-05-26

**Authors:** Stefan Marković, Lucija Levstek, Dušan Žigon, Janez Ščančar, Radmila Milačič

**Affiliations:** ^1^ Department of Environmental Sciences, Jožef Stefan Institute, Ljubljana, Slovenia; ^2^ Jožef Stefan International Postgraduate School, Ljubljana, Slovenia

**Keywords:** chromium speciation, *Taraxacum officinale*, high performance liquid chromatography, isotope dilution inductively coupled plasma mass spectrometry, electrospray ionization high resolution mass spectrometry, laser ablation inductively coupled plasma mass spectrometry

## Abstract

A new analytical procedure for the speciation of chromium (Cr) in plants by high performance liquid chromatography inductively coupled plasma mass spectrometry (HPLC-ICP-MS) was developed using a strong anion-exchange Mono Q column for the separation of the Cr species. To optimize the analytical procedure, Cr complexes were first synthesized from Cr-nitrate with the addition of an excess of ligand (90°C). Cr-oxalate, Cr-malate, Cr-citrate, Cr-aconitate and Cr-quinate complexes and Cr-nitrate (pH 6.5) were chromatographically separated from Cr(VI) by applying linear gradient elution from 100% water to 100% NH_4_Cl at a flow rate of 1.5 ml min^−1^ in 10 min. The column recoveries ranged from 100 to 104%. The exception was Cr-aconitate (column recovery 33%), where a quantitative synthesis was not possible. Good repeatability of the measurements (relative standard deviations better than ± 3%) and low limits of detection (below 0.37 ng ml^−1^ Cr) were achieved for the individual Cr species. The developed analytical procedure was applied to Cr speciation for dandelions (*Taraxacum officinale*) grown in soil with a high Cr content and a study of the uptake and metabolism of Cr species in dandelions grown in soil with a low Cr content treated with solutions of Cr(VI) or Cr-nitrate (5000 ng ml^−1^ Cr, pH 6.5) for 48 h. The separated Cr species were quantified by post-column isotope dilution ICP-MS, while the identification was based on retention times and was also supported by mass spectra obtained with high resolution mass spectrometry (HR-MS). The data indicate that for dandelions grown in Cr-rich soil and that treated with Cr-nitrate (pH 6.5), the Cr was mainly accumulated in the roots, while in plants treated with Cr(VI) (pH 6.5), the Cr was evenly distributed between the roots and the leaves. The Cr species found in dandelion roots and leaves were Cr-aconitate, Cr-malate, and Cr-quinate. The results revealed that Cr(VI) was completely reduced and metabolized to Cr(III) complexes. LA-ICP-MS data showed that the Cr in a leaf of dandelion grown in Cr-rich soil was localized mainly at the apex of the leaf.

## 1 Introduction

Chromium (Cr) and its compounds are important for a variety of industrial processes, e.g., steel making (Sverdrup and Olafsdottir, 2019), leather tanning (Tadesse and Guya, 2017), electroplating (Katirci and Altinsari, 2020), glass and ceramics manufacturing (Yang et al., 2019), treating wood (Tarmian et al., 2020), producing Cr pigments (Yuan et al., 2017) and making vehicles (Chakarova et al., 2018). Cement and its products also contain a relatively high content of Cr (Estokova et al., 2018). All these activities generate large amounts of waste or direct discharges of Cr-rich effluents into the environment (Bhalerao, 2015). Cr from anthropogenic sources can reach environmental compartments in its highly toxic hexavalent [Cr(VI)] form or in the form of the far less toxic trivalent Cr [Cr(III)] compounds (Lay and Levina, 2014; Wang et al., 2017; Sawicka et al., 2021). Hexavalent Cr compounds are much more mobile in the environment than trivalent Cr. Low Cr(III) mobility at neutral pH’s is related to the formation of highly insoluble compounds, mostly oxides and hydroxides (Kimbrough et al., 1999). Cr(VI) is more stable in the alkaline pH range, while in acidic conditions it is rapidly reduced by conventional reducing agents [e.g., iron (II) ions, sulfides, organic matter] or by microorganisms (Ščančar and Milačič, 2014). The uptake of Cr(VI) in plants takes place through the plasma membrane by metabolically actively driven processes *via* the sulfate/phosphate carrier system, while the uptake of Cr(III) is mostly regulated by a passive mechanism without the need for energy (Huang et al., 2018). The accumulation and translocation of Cr in plant depends mainly on its chemical species, which also governs the toxicity of Cr (Sharma et al., 2020). Cr toxicity in plants is manifested in poor seed germination and plant growth, lower yield, mutagenesis, nutrient disorders and the inhibition of photosynthesis and enzymatic processes (Singh et al., 2021).

To prevent environmental pollution, it is necessary to remediate Cr-contaminated sites. Remediations involves physical methods based on adsorption, electrodialysis or ion-exchange, chemical methods based on the reduction of Cr(VI) (Jobby et al., 2018) and nanoremediation procedures (Chen et al., 2020). A promising remediation strategy is the use of living organisms including fungi, algae, bacteria, and plants (Fernández et al., 2018; Chen and Tian, 2021). In order to plan effective phytoremediation strategies and understand Cr detoxification processes, it is necessary to know about the uptake and metabolism of Cr in plants (Bluskov et al., 2005; Shahid et al., 2017; Vidayanti et al., 2017; Srivastava et al., 2021). In such studies, Cr speciation and bio-imaging play a crucial role.

Among the analytical approaches to Cr speciation, high-performance liquid chromatography techniques coupled with inductively coupled plasma mass spectrometry (HPLC-ICP-MS) have the highest selectivity and sensitivity, and generally allow the separation of Cr(VI) from Cr(III) (Novotnik et al., 2012a; Ščančar and Milačič 2014; Hernandez et al., 2018; Caporale et al., 2019; Hamilton et al., 2020). To obtain reliable data for speciation analysis, the specific Cr chemistry associated with its trivalent and hexavalent oxidation states should be considered in each sample matrix (Lay and Levina, 2014). Special attention should also be paid to conditions that might affect the species transformation during sampling, sample pre-treatment and analytical procedure. For this purpose, the use of Cr stable isotopic tracers contributes to the reliability of the analytical results (Novotnik et al., 2013; Zuliani et al., 2013; Novotnik et al., 2015; Séby and Vacchina, 2018; Bergant et al., 2020; Hamilton et al., 2020; Milačič and Ščančar, 2020; Saraiva et al., 2021). An alternative speciation method that provides reliable information about the oxidation states of the Cr is X-ray absorption near-edge structure spectroscopy (XANES). As a non-destructive technique, it is not prone to species interconversion during the analytical procedure. In combination with HPLC-ICP-MS it has been used as a complementary technique for the speciation of Cr in tobacco samples (Cuello et al., 2016).

Speciation procedures based on HPLC-ICP-MS allow reliable and quantitative determinations of Cr(VI), but are generally not specific enough to quantify individual trivalent Cr compounds. Knowledge of the individual Cr(III) species is extremely important to understand the role and behavior of Cr in the environment and living organisms. In living organisms, Cr(III) compounds like positively charged aqua and hydroxo complexes, colloidal Cr(OH)_3_^0^, and negatively charged or neutral Cr(III) complexes with biologically active ligands, show different bioavailabilities. Organic acids are important metabolites of the tricarboxylic acid cycle (TCA, Krebs Cycle) in plants, which is the main energy-producing cycle of the cell (Igamberdiev and Eprintsev, 2016; Jiang et al., 2020). Cr(III) forms different complexes with low molecular mass (LMM) organic acids (Vinokurov and Bondar, 2003). In plants, these complexes are neutral or negatively charged and are difficult to separate on anion-exchange columns. In addition, at physiological pHs, Cr mostly forms two or more complexes with the same ligand, which makes the separation of the Cr species in a mixture of Cr-LMM complexes even more demanding.

In investigations of Cr accumulation and translocation in plants, speciation techniques can be further combined with bio-imaging techniques. To this end, Huang et al. (2018) used XANES and laser ablation (LA)-ICP-MS to study the speciation and localization of Cr in *Coptis chinensis* Franch. Despite the more complete information provided by the combination of the two analytical techniques, such studies have rarely been reported.

In view of the demands for the identification and quantification of Cr(VI) and individual Cr(III) complexes in plants, there is a need to develop new speciation procedures. Reports on such analytical procedures are rare. Therefore, the objectives of our investigation were: 1) to develop a new analytical method for the speciation of Cr-LMM complexes and Cr(VI) in plants using anion-exchange HPLC-ICP-MS; 2) to identify Cr-binding ligands by high resolution mass spectrometry (HR-MS); 3) to use the developed method for Cr speciation in dandelion plants growing in a field treated with tannery waste; 4) to study the uptake and transformation of Cr species in dandelion plants treated with Cr(VI) or Cr-nitrate and 5) to perform bio-imaging by LA-ICP-MS for the localization of Cr in a dandelion leaf.

## 2 Materials and Methods

### 2.1 Instrumentation

The Cr concentrations were determined using ICP-MS on an Agilent (Agilent Technologies Inc., Tokyo, Japan) 7900 instrument. For chromatographic separations, an Agilent series 1200 quaternary pump fitted with a sample injection valve, Rheodyne, model 7725i (Cotati, CA, United States) and 0.1 ml injection loop were used. Cr species were separated on a strong anion-exchange fast protein liquid chromatography (FPLC) column of Mono Q HR 5/5 (GE Healthcare Bio-Sciences, Uppsala, Sweden) (column dimensions 5 mm × 50 mm, stationary phase of polystyrene/divenyl benzene, pH stability 2–12, particle size 10 µm). The column was connected on-line to the ICP-MS instrument. To control the stability of the mass spectrometer, the eluent was spiked (post-column addition) with a rhodium (Rh) internal standard (100 µg l^−1^). Data processing in the speciation analysis was based on the peak area, and was processed using Agilent MassHunter software and OriginPro 2020. The ICP-MS operating parameters were optimized for plasma robustness and for introducing minimum amounts of salts used in the separation procedure with a High Matrix Introduction (HMI) system. To effectively eliminate the polyatomic inferences containing chlorine (on *m/z* 50, 52, and 53), carbon (on *m/z* 52) and sulfur (on *m/z* 50 and 52) the high energy collision mode (HECM), using helium as a collision gas, was applied (Novotnik et al., 2012a). By applying HMI and HECM, the speciation of Cr in matrices high in carbon with moderate concentrations of sulfur (e.g., dandelion plant samples), using NH_4_Cl as the eluent, is possible when Cr 50, 52, and 53 isotopes are followed.

For the Cr mapping of the dandelion leaf, Analyte G2 193 ArF excimer laser ablation with a HelEx II low-dispersion ablation cell (Teledyne Photon Machines Inc., Bozeman, MT, United States) was coupled with an Agilent 8800 ICP-QQQ-MS (Agilent Technologies Inc., Tokyo, Japan) *via* ARIS (Aerosol Rapid Introduction System, Teledyne Photon Machines). The HDIP LA imaging software (purchased from Teledyne Photon Machines) was used to create a Cr distribution map of the sample.

LMM binding ligands in fractions eluted below the chromatographic peaks of Cr were identified “off line” using a hybrid quadrupole high resolution time-of-flight mass spectrometer (Q-ToF Premier from Waters Corp., Manchester, United Kingdom) equipped with an orthogonal Z-Spray source with standard electrospray ionization in the negative ion mode (ESI−).

The ICP-MS, LA-ICP-MS, and ESI-MS operating parameters are provided in Supplementary Table S1.

A CEM Corporation (Matthews, NC, United States) MARS 6 Microwave System was used to digest the dandelion and soil samples.

Binder’s drying and heating oven (Binder GmbH, Tuttlingen, Germany) was used to prepare the Cr-LMM complexes at 90°C.

A universal hot plate IKA RCT basic IKAMAG (IKA-Werke GmbH & Co. KG, Staufen im Breisgau, Germany) was used to prepare the dandelion extracts for speciation analysis.

The pH was measured with a WTW pH meter 3110 (Weilheim, Germany).

A Mettler AE 163 (Mettler Toledo, Zürich, Switzerland) analytical balance was used for weighing.

### 2.2 Reagents and Materials

Ultrapure (Milli-Q) water (18.2 MΩ cm) was obtained from Direct-Q 5 (Millipore Watertown, MA, United States) and was used in all the steps of sample preparation and analysis. Nitric acid (HNO_3_, 65%), hydrochloric acid (HCl, 37%), hydrofluoric acid (HF, 40%) and hydrogen peroxide (H_2_O_2_, 30% solution in water), all of suprapure grade, were obtained from Merck (Darmstadt, Germany). Phosphate buffer prepared from potassium dihydrogen phosphate (KH_2_PO_4_) s.p. and disodium hydrogen phosphate dihydrate (Na_2_HPO_4_ × 2H_2_O) s.p. purchased from Merck was used to adjust the pH. Stock standard solutions of Cr(VI) (K_2_CrO_4_ in water) containing 1000 ± 2 mg l^−1^ of Cr(VI) and Cr(III) solution of Cr-nitrate (1000 ± 2 mg l^−1^ in 5% HNO_3_), both from Merck, were used for the preparation of standard working solutions. Oxalic acid (C_2_H_2_O_4_, Fisher scientific, Loughborough, United Kingdom), malic acid (C_4_H_6_O_5_) and trans-aconitic acid (C_6_H_6_O_6_, Sigma-Aldrich, Steinheim, Germany), citric acid monohydrate (C_6_H_8_O_7_ × H_2_O, VWR chemical international, Leuven, Belgium), and quinic acid (C_7_H_12_O_6_, Merck), all of p.a. grade, were used for the preparation of Cr(III) complexes. Ammonium chloride (NH_4_Cl) s.p. (Merck) was used in the chromatographic separations. Sodium hydroxide (NaOH) p.a., sodium hydrogen carbonate (NaHCO_3_) p.a. and Triton X-100 p.a. obtained from Merck were used for the column cleaning. Formic acid and acetonitrile (both from Sigma-Aldrich) were used in the high resolution mass spectrometry analysis. Rhodium stock solution (1000 ± 2 mg l^−1^ Rh in 8% HCl) purchased from Merck was used as an internal standard in the ICP-MS determinations. Samples were filtered using 0.45 μm Minisart cellulose nitrate membrane filters (Sartorius, Goettingen, Germany). SPS-SW1 Quality Control Material for Surface Water Analysis purchased from SPS Spectrapure Standards AS (Oslo, Norway), the certified reference materials CRM 320R Trace Elements in River Sediment, Community Bureau of Reference (Geel, Belgium) and SRM 1573a Tomato leaves purchased from National Institute of Standards and Technology (NIST), (Gaithersburg, MD, United States) were used to check the accuracy of the total Cr determination in the plants and soil samples. For verifying the accuracy of the determination of Cr(VI) by the HPLC-ICP-MS procedure, Merck’s Certified Reference Material [Chromium Standard Solution 0.050 mg l^−1^ Cr(VI) ± 0.002 mg l^−1^ Cr(VI) K_2_CrO_4_ in H_2_O] was used. The enriched ^50^Cr isotope in the form of ^50^Cr_2_O_3_ was purchased from Oak Ridge National Laboratory (Oak Ridge, TN, United States) and was used for the preparation of the ^50^Cr(VI) isotopic spike solution according to a procedure previously developed in our laboratory (Novotnik et al., 2012b). The composition of the enriched ^50^Cr isotope, declared by producer, was 96.82 ± 0.05% for isotope ^50^Cr, 2.95 ± 0.02% for isotope ^52^Cr, 0.18 ± 0.01% for isotope ^53^Cr and 0.05 ± 0.01% for isotope ^54^Cr. The stability of the LA-ICP-MS measurement was checked with SRM 612 Trace Elements in Glass standard reference material obtained from National Institute of Standards and Technology (NIST). The 0.45 µm nitrocellulose filters (25 mm diameter) obtained from Sartorius were used to prepare the calibration standards in the LA-ICP-MS procedure. SuperFrost Microscope Slides for fixing the sample and the calibration standards were obtained from VWR.

### 2.3 Sampling

Dandelion (*Taraxacum officinale*) roots and the leaves of plants grown in field soil with a high Cr content in the village of Vranja Peč (municipality of Kamnik, Slovenia) and meadow soil with a low Cr content (Jamova cesta, municipality of Ljubljana, Slovenia) were collected in plastic vessels. The soil samples were taken at the same locations as the dandelion plants in double plastic bags. The soil in Vranja Peč was fertilized with tannery waste for 17 years. Sampling of soil and dandelion at this location was performed 37 years after the application of tannery waste.

### 2.4 Sample Preparation for Chromium Speciation Analysis and Determination of Total Chromium Concentrations

#### 2.4.1 Preparation of Phosphate Buffer Solution

435.7 mg of KH_2_PO_4_ were dissolved in 50 ml of water (solution A). 593.5 mg of Na_2_HPO_4_ × 2H_2_O was dissolved in 50 ml of water (solution B). 32.5 ml of solution A was mixed with 17.5 ml of solution B, which gave 0.067 mol l^−1^ phosphate buffer solution with a pH of 6.5.

#### 2.4.2 Preparation of Synthetic Solutions of Cr(III) Complexes, Cr-Nitrate and Cr(VI)

Stock Cr(III) complexes solutions, i.e., Cr-oxalate, Cr-malate and Cr-citrate, Cr-aconitate and Cr-quinate (100 μg ml^−1^ Cr) were made in 10 ml volumetric glass flasks by dissolving an appropriate amount of oxalic, malic, citric, aconitic or quinic acid in water to which 1 ml of Cr-nitrate standard solution (1000 mg l^−1^ Cr) was added. After the flasks were filled with water to the mark, the Cr-to-ligand molar ratio was 1:10 for oxalic, and 1:100 for malic, citric, aconitic, and quinic acids. Stock Cr(III) complexes were formed at 90°C, keeping solutions in a heating oven for 24 h. After cooling to room temperature, working solutions containing 50 ng ml^−1^ Cr, were prepared in 0.013 mol l^−1^ phosphate buffer (pH 6.5, which is similar to the root and leaf sap of the dandelion plant) and left for 4 h at room temperature to stabilize before measurements. Speciation analysis data confirmed that the stock Cr(III) complexes were stable for at least 2 days at room temperature. Cr(VI) and Cr-nitrate (50 ng ml^−1^ Cr) solutions were prepared from stock Cr-nitrate and Cr(VI) solutions in 0.013 mol L^−1^ phosphate buffer (pH 6.5).

#### 2.4.3 Experiments Performed to Study the Uptake of Different Chromium Species Into Plants

To study the uptake and distribution of different Cr species into plants, dandelions grown in soil with a low Cr content were collected in the field. The soil was removed from the roots and washed with water. The rots were immersed for 48 h in solutions containing Cr(VI) or Cr-nitrate with a Cr concentration of 5000 ng ml^−1^. Both solutions were, prepared in 0.134 mol l^−1^ phosphate buffer (pH 6.5). After exposure, the plants were taken from the solutions. Roots and leaves were used for the Cr speciation analysis. In non-exposed plants and in plants after exposure to Cr species, the total Cr content was also determined.

#### 2.4.4 Preparation of Plant Sap Extracts for Speciation Analysis

Dandelion roots and leaves were washed with water and air dried for 8 h. The leaves were separated from the roots and the samples were cut into small pieces with a plastic knife. Approximately 20 g of sample was transferred into 100 ml glass beakers, to which 25 ml of water was added. The beakers were covered with glass lids and left for 6 h on a hot plate (70°C) to extract the sap. Samples were mixed with a glass rod every 30 min. After extraction of the root or leaf sap, the samples were cooled to room temperature. The contents were weighted, filtered through 0.45 µm membrane filters and diluted five times with water before the speciation analysis.

#### 2.4.5 Microwave-Assisted Digestion of Soil and Plant Samples

To determine the Cr content in the soil, approximately 0.2 g of dried (60°C, 48 h) and homogenized sample was transferred into Teflon vessels and 4 ml of HNO_3_, 1 ml of HCl, and 2 ml HF acids were added. The Teflon vessels were capped and subjected to closed-vessel microwave-assisted digestion. After cooling, 12.5 ml of H_3_BO_3_ (4% aqueous solution) was added to dissolve the insoluble calcium fluoride and to complex the excess HF. The contents were again subjected to closed-vessel microwave-assisted digestion and the Cr concentration was determined by ICP-MS (Zuliani et al., 2013).

To determine the Cr content in the plant samples, the roots and leaves were washed with water, cut with a plastic knife and dried at 60°C for 48 h. Approximately 0.2 g of dry samples was transferred into Teflon vessels. Then, 4 ml of s.p. HNO_3_ and 2 ml of s.p. H_2_O_2_ were added and the contents subjected to closed-vessel microwave-assisted digestion. The Cr concentration in the digested samples was determined by ICP-MS (Novotnik et al., 2015).

### 2.5 Analytical Procedure for Speciation of Chromium

0.1 ml of sample was injected onto the column. Linear gradient elution from 100% water to 100% 1 mol l^−1^ NH_4_Cl was applied for 10 min at a flow rate of 1.5 ml min^−1^. The eluate from the column was connected on-line to ICP-MS. After separation, the column was, at the same flow rate, first rinsed for 1 min with water, in the next 2 min with 1% Na_2_CO_3_ in 0.001 mol l^−1^ Triton-X-100, in following 1 min again with water, and then regenerated with 2 mol L^−1^ NH_4_Cl for 3 min. Finally, the column was equilibrated with water for 3 min. The eluents from the regeneration and equilibration of the column were directed to waste. After 10 successive separations of the plant sap samples, it was necessary to clean the column. The cleaning was performed at a flow rate of 1.5 ml min^−1^. The column was first rinsed for 5 min with 1 mol l^−1^ NaOH and then for 20 min with water. This step was repeated twice.

Separated Cr species were quantified using the post-column ID-ICP-MS. Isotopically enriched ^50^Cr(VI) (29.88 ng ml^−1^) was added continuously with a peristaltic pump *via* a T-piece after the separation of Cr species. The mass flow of Cr was plotted versus time during the chromatographic run and the concentrations of Cr species calculated using equations for the post-column species-unspecific ID-ICP-MS analysis (Rodríguez-González et al., 2005).

### 2.6 Preparation of Samples and Calibration Standards for LA-ICP-MS Mapping of Chromium

Dandelion leaves from Vranja Peč were washed with water, left for an hour to dry and attached to a glass slide using a double-sided scotch tape, so that the leaf was completely flat on the tape. The leaf was then left to dry at room temperature for 1 week.

Calibration standards were prepared using 0.45 µm nitrocellulose paper filters. 20 µL of Cr standard in five different concentrations were pipetted into the center of the filters and allowed to air dry for 5 h. After drying, a 5 mm circle was cut out of the center of the filter paper with a hollow hole punch. This was done to minimize the uneven distribution of Cr throughout the filter paper due to the “coffee stain” effect. Six cutouts (blank and five Cr standards) were weighted and transferred to a 10 ml graduated polyethylene tube. Next, 0.2 ml of HNO_3_ and 0.2 ml of H_2_O_2_ were added and the tubes were sealed with caps and Teflon tape. The samples were left at 80°C overnight to digest the contents. Each tube was then filled to 2 ml with water and weighed, and the Cr concentration in the dissolved filters was determined by ICP-MS. Parallel six filter cutouts for each Cr concentration level were attached on a glass slide using a double-sided scotch tape so that all the Cr calibration standards were present on one slide.

### 2.7 Analytical Procedure for Laser Ablation Imaging

The laser beam was a 35 µm square-shaped spot, which provided an even energy delivery with 3.5 J cm^2^ fluency. Its repetition rate was 50 Hz, while the scan speed was 200 µm s^−1^ with connected parallel lines. The total dwell time on the ICP-MS was 0.2 s per cycle. ARIS was used for a rapid analysis with good signal retention. Helium carrier gas (0.6 l min^−1^) was used to introduce a sample aerosol into the ICP. Argon make-up gas (0.95 l min^−1^) was introduced through the ARIS mixing bulb. The washout time of a single laser pulse with the measurement parameters described above was 58 ms. The LA-ICP-MS system was tuned daily using NIST SRM 612 glass standard reference material. HDIP software was used for the LA-ICP-MS imaging.

### 2.8 Analytical Procedure for Mass Spectrometric Analysis

10 µL of sample was injected directly through the electrospray ion source into the high resolution tandem Q-ToF mass spectrometer at a mass resolution of 10,000. Mass spectra were recorded in the negative ion mode in the mass range *m/z* 50 to *m/z* 1000.

If not stated otherwise, all the analyses were made in duplicate.

## 3 Results and Discussion

### 3.1 Development of the HPLC-ICP-MS Procedure for Speciation of Cr(III) Complexes, Cr(VI) and Ionic Cr(III)

Plants are rich in LMM organic acids, which readily form complexes with Cr(III). Most of them derive from the TCA cycle. Citrate is formed by the citrate synthase reaction from glucose. From citrate, cis-aconitate and trans-aconitate are formed in enzymatic reactions. Trans-aconitate, which is also produced by citrate dehydrase, is a more stable isomer and accumulates in plants (Igamberdiev and Eprintsev, 2016). Takiguchi et al. (2021) demonstrated that trans-aconite can also be produced by bacteria with a biosynthetic pathway. In another path of the TCA cycle, malate and oxalate are formed by several enzymatic reactions (Igamberdiev and Eprintsev, 2016). Quinic acid is also widespread in the plant kingdom. It is a key intermediate in the biosynthesis of aromatic compounds in living systems, arising from phenylpropanoid (Magaña et al., 2021). Due to their prevalence in plants and because they form moderate-to-strong complexes with Cr(III), citric, aconitic, oxalic, malic, and quinic acids were selected to prepare LMM-Cr(III) complexes for the Cr speciation study.

The potential of the separation of ionic Cr(III) (Cr-nitrate) and Cr(VI) was previously investigated in our group, using a strong anion-exchange MonoQ column. At physiological pH values (pH 6.0–7.5), ionic Cr(III) is present mainly as colloidal Cr(OH)_3_ and is strongly adsorbed on the stationary phase of the anion-exchange column, while Cr(VI), as a negatively charged species, is retained by the column resin and is eluted with the increasing ionic strength of the eluent (NaCl) (Novotnik et al., 2012a).

To achieve the best selectivity and repeatability of the separation of Cr-oxalate, Cr-malate, Cr-citrate, Cr-aconitate, Cr-quinate, Cr-nitrate, and Cr(VI) on the anion-exchange HPLC column, in the present investigation, parameters like sample volume, flow rate, and eluent composition were optimized using synthetic solutions of Cr(III) complexes, Cr-nitrate and Cr(VI) (for preparation see Section 2.4.2). First, the most suitable eluent was chosen. For this purpose, NaCl and NH_4_Cl solutions were tested. Due to the better selectivity, the latter was further selected in the optimization procedure. The optimal separation of Cr species was obtained when 0.1 ml of sample was injected onto the column and linear gradient elution from 100% water to 100% 1 mol l^−1^ NH_4_Cl was applied in 10 min at a flow rate of 1.5 ml min^−1^. For effective column cleaning, 1% Na_2_CO_3_ in 0.001 mol l^−1^ Triton-X-100 was first applied to wash the plant sap constituents adsorbed on the column resin (parts of the cell walls membranes), while 2 mol l^−1^ NH_4_Cl was used for the column’s regeneration. The analytical procedure for the speciation of Cr is provided in Section 2.5.

**FIGURE 1:**
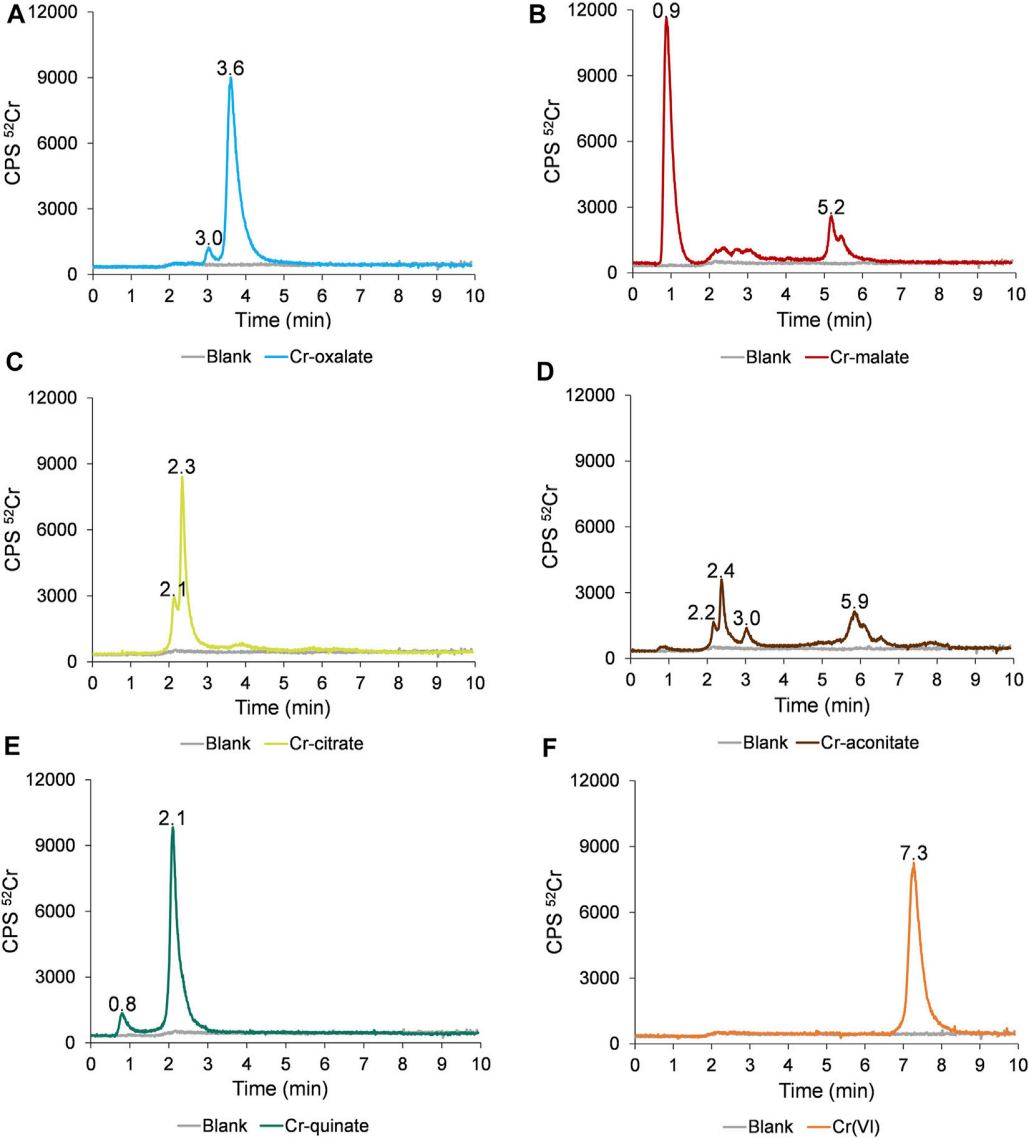
Speciation of Cr in synthetic solutions of **(A)** Cr-oxalate, **(B)** Cr-malate, **(C)** Cr-citrate, **(D)** Cr-aconitate, **(E)** Cr-quinate, and **(F)** Cr(VI), containing 50 ng ml^−1^ Cr, prepared in 0.013 mol l^−1^ phosphate buffer (pH 6.5) applying the HPLC-ICP-MS procedure.

The separation of the Cr species in synthetic solutions of Cr-oxalate, Cr-malate, Cr-citrate, Cr-aconitate, Cr-quinate, and Cr(VI) at pH 6.5, applying the HPLC-ICP-MS procedure, is presented in Figure 1.

The data in Figure 1F indicate that Cr(VI) is eluted from 6.8 to 8.2 min and is separated from the Cr(III)-LMM complexes. The Cr(VI) and Cr(III)-LMM complexes are also resolved from Cr(III)-nitrate, which is strongly adsorbed on the stationary phase of the column (Supplementary Figure S1), while only a negligible peak of ionic Cr(III) is eluted with the solvent front.

From Figure 1A it is further evident that Cr-oxalate is mainly eluted as a very small peak from 2.9 to 3.3 min and as a major peak from 3.3 to 4.5 min. Ciavatta et al. (2000) calculated stability constants for Cr-oxalate complexes. At physiological pHs, [Cr(ox)]^−^ and [Cr(ox)_3_]^3−^ complexes exist. Their reported log *K* values are 13.54 and 18.07, respectively. Theoretical calculations of Ciavatta et al. (2000) support the presumption that the major Cr-oxalate peak eluted from the column (3.3–4.5 min), corresponds to [Cr(ox)_3_]^3−^ and a small one (2.9–3.3 min) to the [Cr(ox)]^−^ complex.

Cr-malate (Figure 1B) is eluted as a dominant peak with the solvent front from 0.7 to 1.4 min, as a small, broadened peak from 2.0 to 3.2 min and as a larger peak from 5.0 to 5.6 min. Based on data from the potentiometric titrations, Hamada et al. (2013) proposed the formation of mixed hydroxo-malate [Cr(mal)(OH)]^−^ and the dimeric [Cr_2_(mal)_2_(OH)_3_]^3−^ complexes as major Cr-malate species. The authors also did not exclude the possibility of oligomeric complexes being formed. However, no data were provided on the stability constants of these Cr-malate complexes. The only general data on the complexation constant of Cr(III) with malic acid, which is 5.4, was reported by Mo et al. (2018). On the basis of our experimental data and the incidence of Cr-malate complexes proposed by Hamada et al. (2013), we can conclude that the first Cr-malate peak (0.7–1.4 min), which is weakly bound to the column resin, most likely corresponds to [Cr(mal)(OH)]^−^, the broadened Cr-malate peak (2.0–3.2 min) to the mixture of the Cr-malate oligomeric complexes, while that eluted from 5.0 to 5.6 min, to the [Cr_2_(mal)_2_(OH)_3_]^3−^ complex. To prove that the first eluted Cr peak is the Cr-malate complex, and not ionic Cr, the speciation of Cr was also performed with a solution of ionic Cr(III) and Cr-malate at the pH 3.5 and 6.5. These results are summarized in Supplementary Figure S2. At pH 3.5, ionic Cr elutes with the solvent front as a positively charged Cr^3+^ species and is almost overlapped with the [Cr(mal)(OH)]^−^ complex. With the increasing pH, ionic Cr [colloidal Cr(OH)_3_] is adsorbed on the stationary phase of the column, while [Cr(mal)(OH)]^−^ elutes with a solvent front, confirming the presence of the Cr-malate complex.

As can be seen from Figure 1C, Cr-citrate is mostly eluted as two non-resolved peaks from 1.9 to 3.0 min. Gabriel et al. (2007), who performed theoretical speciation of Cr-citrate complexes in aqueous solutions, reported that at physiological pHs, [Cr(cit)OH]^−^ and [Cr(cit)_2_OH]^2−^ dominate, while their calculated log *K* are 2.56 and 6.13, respectively. The theoretically calculated speciation data supported the presumption that the Cr species eluted from the column correspond to weak [Cr(cit)OH]^−^ and moderately stable [Cr(cit)_2_OH]^4−^ complexes.

Cr-aconitate (Figure 1D) elutes in several peaks: the sharpest from 2.1 to 2.9 min, a very small peak from 2.9 to 3.2 min and a larger one from 5.6 to 6.6 min. There are no studies in the scientific literature reporting species distribution diagrams of Cr-aconitate complexes or their stability constants. Therefore, it is difficult to predict which Cr-aconitate complexes are eluted from the column resin (Figure 1D). The only report available refers to Cd-aconitate complexes that are very stable. At the physiological pHs, the [Cd_3_(aconit)_2_]^0^ complex prevails with a corresponding log *K* of 15.01 (Κostakis et al., 2009). Thus, it can be assumed that Cr can also form strong complexes with aconitic acid.

Finally, the elution profile of Cr-quinate (Figure 1E) shows a small peak eluted with the solvent front and the major one, eluted from 1.9 to 2.9 min. According to a study of the occurrence of Cr-quinato complexes in the aqueous solutions performed in Salifoglou’s group (Mateescu et al., 2013), at physiological pHs the most stable complex is [Cr_3_(quin)_6_(OH)_3_]^0^ with the corresponding log *K* of 10.02, while the complex [Cr(quin) (OH)]^+^ with log *K* of −0.94 is a relatively unstable species. Therefore, we can conclude that the Cr peak eluted from 1.8 to 3 min corresponds to the strong [Cr_3_(quin)_6_(OH)_3_]^0^ complex, and the small one, eluted with a solvent front, to the [Cr(quin)(OH)]^+^ complex.

**FIGURE 2:**
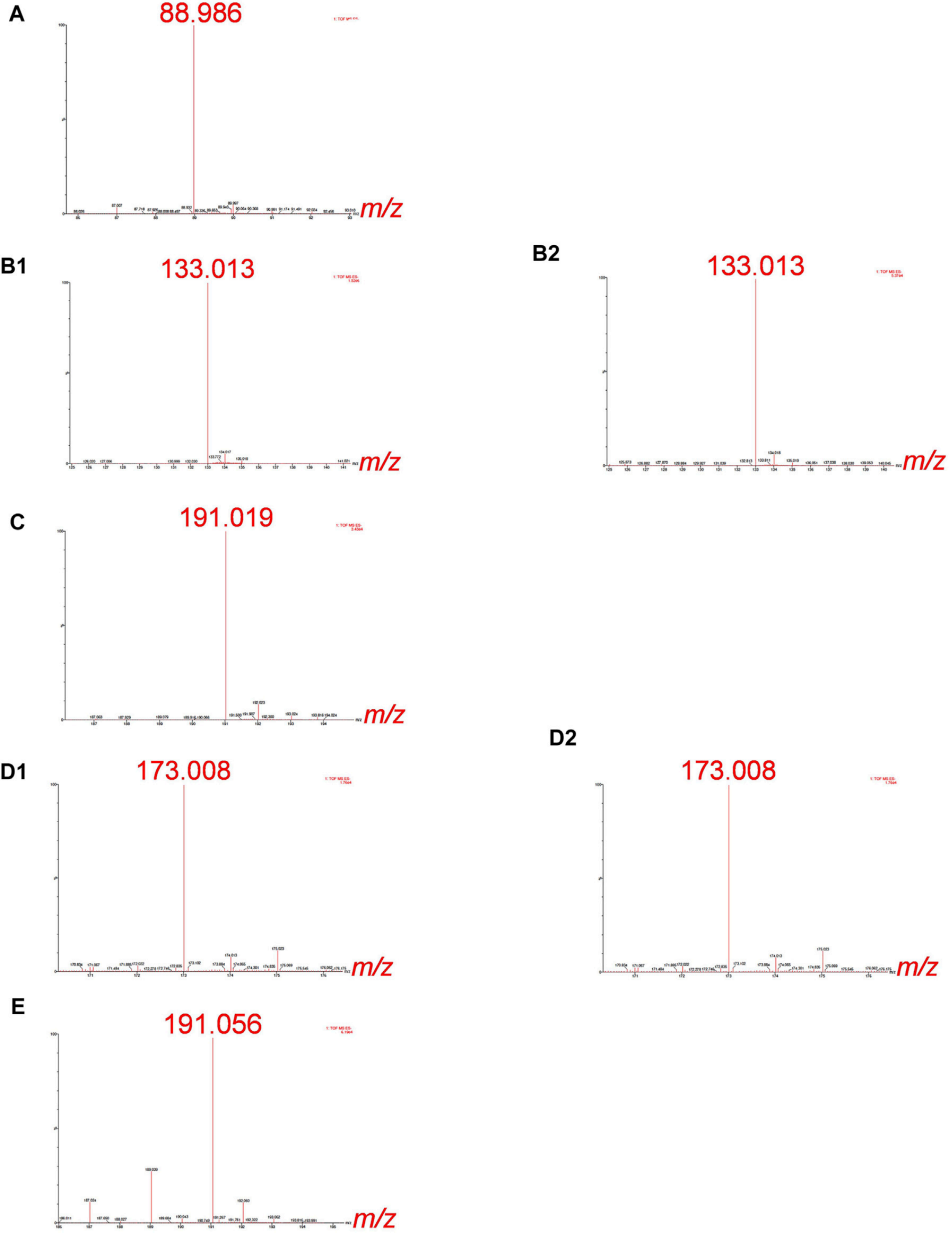
HR-MS spectra of the Cr species eluted under the chromatographic peaks of synthetic solutions of **(A)** Cr-oxalate (3.3–4.5 min), **(B1)** Cr-malate (0.7–1.4 min), **(B2)** Cr-malate (5.0–5.6 min), **(C)** Cr-citrate (1.9–3.0 min), **(D1)** Cr-aconitate (2.1–2.9 min), **(D2)** Cr-aconitate (5.6–6.6 min), and **(E)** Cr-quinate (1.9–2.9 min). 10 µg ml^−1^ Cr, Cr-to-ligand molar ratio for Cr-oxalate 1:10, and for Cr-malate, Cr-citrate, Cr-aconitate, and Cr-quinate 1:100, prepared in 0.013 mol l^−1^ phosphate buffer (pH 6.5).

For the Cr-binding ligands’ identification by ESI-Q-ToF, synthetic solutions of Cr-LMM complexes were injected into the column and the fractions eluted below the chromatographic peaks of Cr collected. The mass spectra were recorded in the negative ion mode in the mass range *m/z* 50 to *m/z* 1000, identifying the mass of the deprotonated molecular ions [M−H]^−^. The high-resolution MS measurements of these experiments are provided in Figure 2.

The HR-MS measurement of the fraction eluted below the Cr-oxalate peak (Figure 2A), gave an accurate mass at 88.986, corresponding to the elemental composition of C_2_HO_4_ (deprotonated oxalic acid). This indicates that the Cr species eluted from the column from 3.3 to 4.5 min belongs to Cr-oxalate complex. In the fractions of the Cr-malate complexes eluted from the column from 0.7 to 1.4 min and 5.0–5.6 min (Figures 2B1, B2) the HR-MS measurements gave an accurate mass at 133.013, which corresponds to the elemental composition of C_4_H_5_O_5_ (deprotonated malic acid). This confirms that Cr species eluted below Cr peaks belong to the Cr-malate complexes. From Figure 1C it can be further seen that the HR-MS measurement of the fraction eluted below the Cr-citrate peak (1.9–3 min) gave an accurate mass at 191.019, corresponding to the elemental composition of C_6_H_7_O_7_ (deprotonated citric acid). This proves that Cr species eluted from the column belong to the Cr-citrate complexes. In fractions of Cr-aconitate complexes eluted from the column from 2.1 to 2.9 min and 5.6–6.6 min (Figures 2D1, D2), HR-MS measurements gave an accurate mass at 173.008, which corresponds to the elemental composition of C_6_H_5_O_6_ (deprotonated aconitic acid). This confirms that the Cr species eluted below the Cr peaks belong to the Cr-aconitate complexes. Finally, the HR-MS measurement of the fraction eluted below the Cr-quinate peak (Figure 2E), gave an accurate mass at 191.056, corresponding to the elemental composition of C_7_H_11_O_6_ (deprotonated quinic acid). This indicates that Cr species eluted from the column from 1.9 to 2.9 min belongs to the Cr-quinate complex. Furthermore, it should be noted that the HR-MS measurements allow a reliable distinction between the deprotonated molecular ions of citric and quinic acids of similar masses. Namely, their respective *m/z* 191.019 and *m/z* 191.056 are well resolved

The possibility of separating Cr(III) complexes on a column and the use of complementary HR-MS measurements to identify the Cr-binding ligands, importantly extends the use of the developed analytical method to the speciation of Cr in plant samples.

### 3.2 Figures of Merits for the Developed Analytical Procedure

The column recovery was tested by applying the speciation analysis of synthetic solutions of Cr(III)-LMM complexes and Cr(VI) with a concentration of 50 ng ml^−1^ Cr. For this purpose, fractions below the chromatographic peaks containing Cr were collected and the Cr content in them determined by ICP-MS. Column recoveries were calculated as the ratio between the Cr concentration determined in the fractions eluted and the Cr concentration injected into the column. The results are summarized in Table 1.

**TABLE 1:** Column recoveries, repeatability of measurements based on mean of CPS area of 6 consecutive separations, LODs and LOQs for Cr-oxalate, Cr-malate, Cr-citrate, Cr-aconitate, and Cr(VI) (Cr concentration 50 ng ml^−1^, pH 6.5) by the HPLC-ICP-MS procedure.

Cr species	Cr added (ng ml^−1^)	Cr found (ng ml^−1^)	Column recovery (%)	Repeatability of measurement (mean of CPS area, *n* = 6)	RSD between consecutive separations (%)	LOD (ng mL^−1^ Cr)	LOQ (ng mL^−1^ Cr)
Cr-oxalate	50.0 ± 0.5	49.8 ± 1.5	100	148953	3.0	0.35	1.16
Cr-malate	50.0 ± 0.5	52.0 ± 1.5	104	243368	2.5	0.32^a^	1.07^a^
						0.35^b^	1.17^b^
Cr-citrate	50.0 ± 0.5	51.0 ± 1.5	102	191617	2.9	0.34	1.13
Cr-aconitate	50.0 ± 0.5	16.3 ± 0.5	33	53252	3.1	0.35^c^	1.16^c^
						0.36^d^	1.20^d^
Cr-quinate	50.0 ± 0.5	52.1 ± 1.5	104	204000	3.0	0.33	1.10
Cr(VI)	50.0 ± 0.5	50.1 ± 1.5	100	212516	2.6	0.37	1.23

± uncertainty of measurement.

a Cr-malate 0.7–1.6 min.

b Cr-malate 5.0–5.8 min.

c Cr-aconitate 2.0–2.9 min.

d Cr-aconitate 5.6–6.6 min.

The Cr-oxalate, Cr-malate, Cr-citrate, Cr-quinate and Cr(VI) were quantitatively eluted from the column (column recoveries ranged between 100 and 104%). The exception was Cr-aconitate, for which it was not possible to quantitatively synthesize the complex. Consequently, the column recovery was only 33%, while ionic Cr(III), which was not complexed with aconitic acid, was at pH 6.5, strongly adsorbed on the stationary phase of the column. Nevertheless, the proportion of Cr eluted in the form of Cr-aconitate is a good representation of its behavior on the column. In plants, Cr is not present in a form of free ionic species, but it is complexed with the available LMM organic acids. Thus, in real plant samples, the proportion associated with Cr-aconitate can still be accurately quantified using the current speciation procedure.

The repeatability of the measurements was verified by performing six consecutive speciation analyses of synthetic solutions of Cr-oxalate, Cr-malate, Cr-citrate, Cr-aconitate, Cr-quinate, and Cr(VI), containing 50 ng ml^−1^ Cr. The data are provided in Table 1. Good repeatability of the measuremenst was obtained. The relative standard deviations (RSDs) between consecutive separations for the separated Cr(III)-LMM complexes and Cr(VI) ranged between ±2.5% and ±3.1%.

The limits of detection (LODs) and quantification (LOQs) for the determination of the Cr(III)-LMM complexes and the Cr(VI) were calculated as the concentrations that provided a signal (peak area) equal to 3*s* or 10*s* of the blank sample (0.013 mol l^−1^ phosphate buffer, pH 6.5) in the chromatogram, respectively. To calculate the LODs and LOQs, eight blank samples were injected, and the speciation procedure applied (Table 1).

The LODs and LOQs ranged from 0.32 to 0.37 ng ml^−1^ Cr and from 1.07 to 1.23 ng ml^−1^ Cr, respectively. Such LOQs are low enough to perform Cr speciation analysis in plant samples.

The linearity of the measurement for the separated Cr species was confirmed over the concentration range from LOQ to 500 ng ml^−1^ Cr, with a correlation coefficient (*R* ^*2*^) better than 0.997.

The accuracy of the determination of Cr(VI) was tested by analyzing the CRM Chromium Standard Solution in H_2_O. The determined Cr(VI) (46.2 ± 1.1 ng ml^−1^) agreed well with the certified value (47 ± 2 ng ml^−1^), confirming the accuracy of the HPLC-ICP-MS speciation procedure used. Since there are no certified reference materials available for Cr(III)-LMM complexes, the accuracy of the determination of the Cr species in plant samples was checked by the speciated ID-ICP-MS procedure. Moreover, speciated ID-ICP-MS was used for quantification of separated Cr(III) species, as this is the most accurate and reliable calibration approach and does not require compound-based standards. In addition, no standards are available for individual Cr(III) complexes.

### 3.3 Speciation of Chromium in Dandelion (*Taraxacum officinale*)

The developed analytical procedure was applied to the speciation of Cr in root and leaf samples of dandelion plants grown in field soil from Vranja Peč with a high Cr content (581 ± 11 mg kg^−1^ Cr) and in the study on the uptake and distribution of Cr species in plants treated with Cr(VI) or Cr-nitrate. These plants were grown in the soil with a low Cr content (55.9 ± 1.2 mg kg^−1^). The total Cr content in the dandelion roots and leaves of plants from Vranja Peč was 18.5 ± 0.8 mg kg^−1^ and 1.12 ± 0.05 mg kg^−1^, respectively, while the dandelion roots and leaves of the plants grown in soil with a low Cr content [before treatment with Cr(VI) or Cr-nitrate] was 3.5 ± 0.2 mg kg^−1^ and 0.32 ± 0.02 mg kg^−1^, respectively. All the Cr concentrations in the soil or plant samples were calculated on a dry weight (d.w.) basis.

The accuracy of the total Cr determination by ICP-MS was verified by analysing CRM SPS-SW1 surface water, CRM 320R and SRM 1573a. The determined Cr concentrations agreed well with the certified values (see data in Supplementary Table S2), confirming that the analytical procedures applied for the determination of the total Cr concentrations were accurate.

#### 3.3.1 Speciation of Chromium in the Dandelion Plant From Vranja Pe

Dandelion root and leaf sap samples of a plant grown in Vranja Peč in the soil with a high Cr content were prepared according to the procedure described in Section 2.4.4. Before the speciation analysis, the presence of LMM organic acids was checked by ESI-Q-ToF mass spectrometry. In Supplementary Figure S3 HR-MS spectra in the mass range *m/z* 50 to 200 are presented. In the root and leaf sap, *m/z* 133.013 and its dehydrated fragment *m/z* 115.003 prevail, demonstrating that malic acid is the major ligand that can bind Cr. Furthermore, *m/z* 149.008, indicates the presence of tartaric acid in the dandelion sap samples. However, at physiological pHs, tartaric acid forms weak complexes with Cr. The log *K* values for [Cr(tart)]^−^ and [Cr(tart)_3_]^3−^ are 2.0 and 3.59, respectively (Sharma et al., 1985). Therefore, Cr is unlikely to be complexed with tartaric acid in the dandelion plant sap, since other LMM binding ligands form much more stable Cr-LMM complexes (see Section 3.1). In the root and leaf sap samples, *m/z* 191.019 and *m/z* 191.056 of similar peak intensity, and *m/z* 173.008 of slightly lower peak intensity were also identified, confirming the presence of citric, quinic and aconitic acids. The later LMM organic acids can bind the Cr in moderately stable or strong Cr complexes.

**FIGURE 3:**
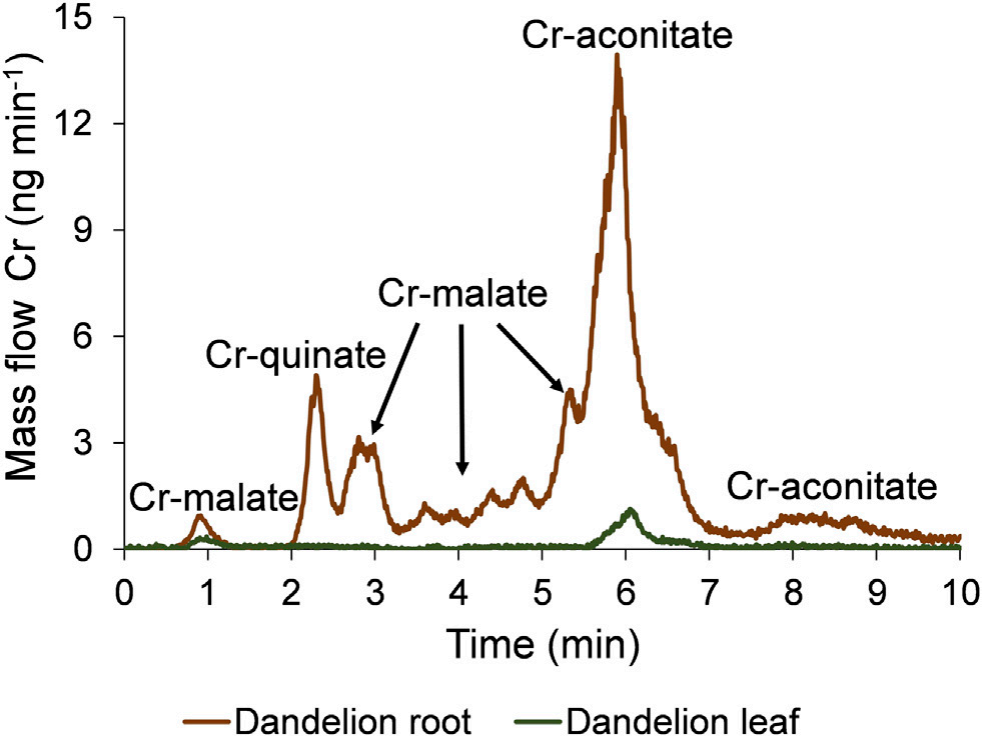
Speciation of Cr in five-times diluted dandelion root and leaf sap by HPLC-ICP-MS. Cr mass flow is based on measurements of isotope ratios *m/*z 50 and 52. Dandelions were grown in soil from Vranja Peč with a high Cr content (581 ± 11 mg kg^−1^).

**FIGURE 4:**
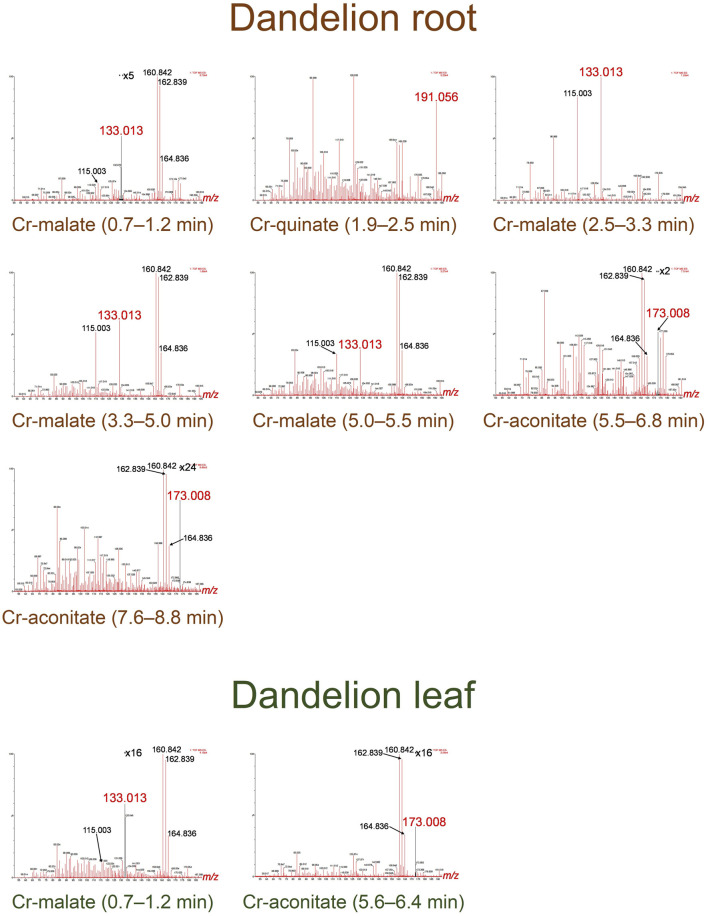
HR-MS spectra of the Cr species eluted under the chromatographic peaks of dandelion root and leaf sap from Vranja Peč.

The speciation of Cr in the dandelion root and leaf sap in five-times diluted samples using the developed HPLC-ICP-MS procedure and the corresponding HR-MS spectra of the Cr species eluted under the chromatographic peaks are presented in Figures 3, 4.

To quantify the separated Cr species with the post-column ID-ICP-MS, isotopes at *m/z* 50 and 52 were monitored. The mass flow of ^52^Cr was plotted versus time throughout the chromatographic run (Figure 3). Data from Figure 3 show that the Cr in dandelion root sap is eluted in multiple peaks, while in dandelion leaf sap, two well separated peaks were detected.

By comparing the elution times of the Cr species in the dandelion root sap: 0.7–1.2 min, 2.5–3.3 min, 3.3–5.0 min and 5.0–5.5 min (Figure 3) with elution times of the Cr-complexes (Figure 1), it can be assumed that these Cr peaks correspond to the Cr-malate. This assumption was further confirmed by HR-MS measurements of the Cr-binding ligands below the Cr peaks (Figure 4), indicating the presence of a deprotonated molecular ion 133.013. Thus, it can be concluded that the four above Cr peaks correspond to the Cr-malate. The Cr peak eluted from 1.9 to 2.5 min (Figure 3) can be associated with both the Cr-citrate and Cr-quinate complexes (see elution times of Cr-complexes, Figure 1). Since citric and quinic acids are present in similar concentrations in plant sap (Figure 2), the formation of the Cr-quinate complex is more likely, because the Cr-quinate complex is much more stable than Cr-citrate (log *K* are 10.02 and 6.13, respectively). This assumption was also confirmed by the HR-MS measurements of the peak eluted in the root sap from 1.9 to 2.5 min (Figure 4), with only the presence of deprotonated quinic acid detected (*m/z* 191.056). Based on the elution time of the major Cr peak from 5.5 to 6.8 min and the broadened peak from 7.6 to 8.8 min (Figure 3), and the HR-MS measurements of the Cr-binding ligands that identified the deprotonated molecular ion 173.008, it can be concluded that the Cr species in these two peaks are associated with Cr-aconitate.

In the dandelion leaf sap, Cr species eluted from 0.7 to 1.2 min and from 5.6 to 6.4 min, most likely correspond to Cr-malate and Cr-aconitate, respectively (Figure 3). This statement was further confirmed by the HR-MS measurements (Figure 4), which indicate the presence of deprotonated malic and aconitic acid below the two eluted Cr peaks.

Moreover, the HR-MS measurements of the Cr species eluted from 0.7 to 1.2 min in dandelion root and leaf sap (Figure 4) indicate the presence of an isotopic pattern of the chlorine atom (*m/z* 160.842, 162.839, and 164.836). The chlorine, which is coeluted with the Cr-malate peak (from 0.7 to 1.2 min), arises from the chloride present in the root and leaf sap. The same isotopic pattern of chlorine is also observed in all the Cr peaks eluted from 3.3 to 8.8 min. This chlorine is introduced with NH_4_Cl eluent used in the separation procedure (gradient elution from 100% water to 100% 1 mol l^−1^ NH_4_Cl in 10 min). In Cr-containing fractions, other small peaks observed in the HR-MS measurements originate from the root or leaf sap constituents.

In the chromatographic peaks eluted from the column, the Cr concentrations were further quantified by post-column ID-ICP-MS (2.5.1). These results are presented in Table 2. When Cr-malate or Cr-aconitate complexes were eluted in multiple peaks, the concentrations of the Cr-malate or Cr-aconitate complexes in Table 2 represent the sum of their individual Cr species separated during the chromatographic run. Table 2 also shows the sum of the Cr species concentrations, the total Cr concentrations and the ratio between the sum of the Cr species concentrations and the total Cr concentrations in dandelion root and leaf sap.

**TABLE 2:** Concentrations of Cr species, sum of Cr species concentrations and total Cr concentrations in dandelion root and dandelion leaf sap from dandelions grown in soil from Vranja peč with a high Cr content 581 mg kg^−1^ and dandelion grown in soil with a low Cr content 55.9 mg kg^−1^, exposed for 48 h to synthetic solutions of Cr(VI) and Cr-nitrate (pH 6.5, 5000 ng ml^−1^ Cr). Total Cr concentrations were determined by ICP-MS, while concentrations of Cr species were determined by post-column ID-ICP-MS. Concentrations are expressed on a d.w. basis. Measurement uncertainty for ICP-MS is better than ±2%, while for post-column ID-ICP-MS ± 3%.

Dandelion sap	Concentrations of Cr species			Sum of Cr species concentrations	Total Cr concentrations	Sum of Cr species concentrations/Total Cr concentrations
sample	Cr-quinate	Cr-malate	Cr-aconitate			
	(mg kg^−1^)	(mg kg^−1^)	(mg kg^−1^)	(mg kg^−1^)	(mg kg^−1^)	(%)
Vranja peč root	0.677	2.65	5.10	8.42	12.46	67.6
Vranja peč leaf	<0.05	0.154	0.438	0.592	0.782	75.7
Exposure to Cr(VI)—root	3.13	12.7	17.2	33.1	47.53	69.6
Exposure to Cr(VI)—leaf	0.804	29.3	5.37	35.5	52.87	67.2
Exposure to Cr-nitrate—root	1.09	6.63	8.38	16.1	27.22	59.1
Exposure to Cr-nitrate—leaf	0.131	2.68	4.36	7.16	9.50	75.4

As can be seen, Cr-aconitate (5.10 mg kg^−1^) is the dominant Cr species in the dandelion root sap from Vranja Peč, while Cr-malate and Cr-quinate are present in lower concentrations (2.65 and 0.677 mg kg^−1^, respectively). The sum of the Cr species eluted from the column represent 68% of the total Cr content in the root sap. The residual portion of Cr was adsorbed to the column stationary phase and was rinsed from the column during the cleaning procedure. Presumably, this fraction of Cr is strongly bound to cell walls and was not removed by filtration of the root sap. In the dandelion leaf sap from Vranja Peč, Cr-aconitate and Cr-malate were found in significantly lower concentrations than in the root sap (0.438 and 0.154 mg kg^−1^, respectively). About 25% of the Cr in the leaf sap remained in the cell walls and was adsorbed by the column stationary phase.

The developed speciation procedure in combination with the HR-MS revealed that Cr speciation is also possible in real plant samples. Although the elution profiles of the Cr-citrate and Cr-quinate complexes overlap, the HR-MS measurements in the root sap confirm the presence of only Cr-quinate. Quinic acid, one of the important LMM organic acids found in coffee beans, tea leaves and medicinal plants (Marques and Farah, 2009), also exists in the dandelion. In plant sap, where quinic and citric acids are present in similar concentrations, quinic acid preferably binds the Cr.

To the best of our knowledge, such an advanced speciation analysis of Cr in plants, which would allow the quantitative determination of Cr-LMM complexes in plant samples, has not yet been performed. Furthermore, the developed methodology also enables the study of the uptake and transformation of Cr species in plants.

#### 3.3.2 Uptake and Metabolism of Chromium in Dandelion Exposed to Synthetic Solutions of Cr(VI) and Cr-Nitrate

**FIGURE 5:**
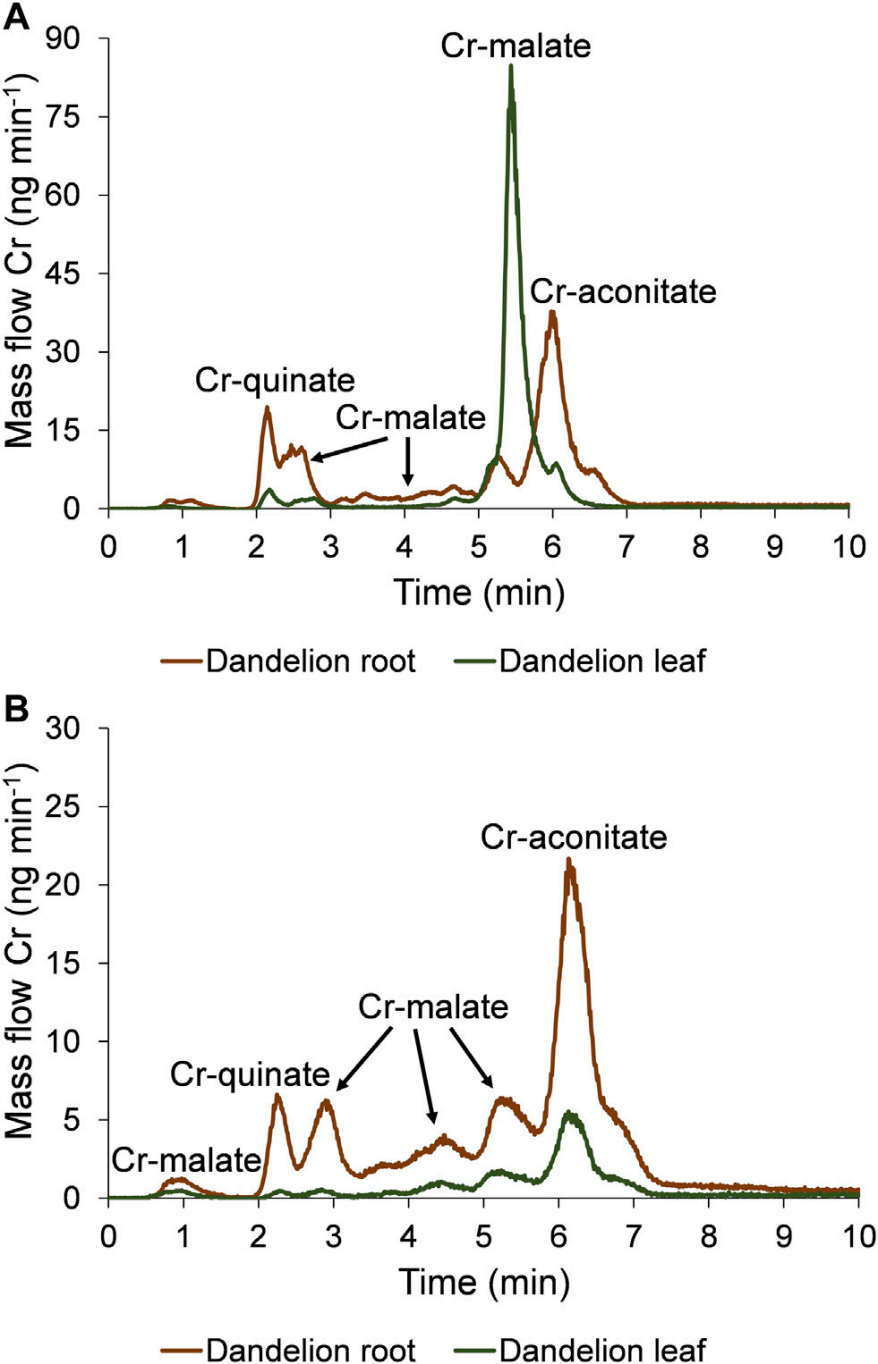
Speciation of Cr in five-times diluted dandelion root and leaf sap by HPLC-ICP-MS. Cr mass flow is based on measurements of isotope ratios *m/*z 50 and 52. Dandelion was grown in soil with a low Cr content (55.9 ± 1.2 mg kg^−1^), and was exposed for 48 h to synthetic solutions of **(A)** Cr(VI) and **(B)** Cr-nitrate (pH 6.5, 5000 ng ml^−1^ Cr).

The uptake and metabolism of Cr species in dandelion roots and leaves exposed for 48 h to synthetic solutions of Cr(VI) and Cr-nitrate (5000 ng ml^−1^, pH 6.5) were investigated by speciation analysis in combination with HR-MS measurements. Experiments were performed according to the procedure described in Section 2.4.3. The results of the speciation analysis are presented in Figure 5, and the corresponding HR-MS measurements in Supplementary Figures S4, S5.

Data from Figure 5A indicate that after the exposure of the dandelion plant to the solution of Cr(VI), Cr was completely reduced to Cr(III) in the dandelion root and complexed with LMM organic acids. Based on the elution times of the Cr peaks, and the HR-MS measurements of the ligands that complex Cr in the root sap (Supplementary Figure S4), it can be concluded that the Cr species associated with the chromatographic peak eluted from 1.9 to 2.5 min corresponds to Cr-quinate, the peaks eluted from 2.5 to 3.0 min, 3.0–4.9 min and 4.9–5.5 min correspond to Cr-malate, while the major peak eluted from 5.5 to 7.0 min corresponds to Cr-aconitate. The same Cr species were translocated from root to leaf, with Cr-malate being the predominant Cr species. Based on data from Figure 5A and Supplementary Figure S4 it can be concluded that in the leaf sap, the peak eluted from 1.9 to 2.5 min corresponds to Cr-quinate, a small peak from 2.5 to 3.0 min and the most intensive peak from 5.0 to 5.8 min corresponds to Cr-malate, while a small peak from 5.8 to 6.3 min corresponds to Cr-aconitate.

The fact that Cr(VI) cannot exist in plants has been reported by many researchers who have used an appropriate analytical methodology for Cr speciation (Milačič and Ščančar, 2020). The ability of plants to completely reduce Cr(VI) was also observed by Hamilton et al. (2020), who studied the uptake of Cr(VI) into the root systems of *Spinacia oleracea*. With the use of enriched isotopes of ^50^Cr(III) and ^53^Cr(VI) and speciation analysis, the authors proved that Cr(VI) was completely reduced to Cr(III). However, using the applied analytical methodologies (Hamilton et al., 2020; Milačič and Ščančar, 2020) it was not possible to determine any Cr(III) complexes present in the plant sap.

When dandelion was exposed to Cr-nitrate solution (Figure 5B, Supplementary Figure S5), Cr-malate was eluted in peaks from 0.7 to 1.2 min, 2.5–3.3 min, 3.3–4.9 min, and 4.9–5.7 min, Cr-quinate in a peak from 1.9 to 2.5 min, and Cr-aconitate, which was the most intensive Cr peak, from 5.7 to 7.2 min. The same Cr species were also transferred to the leaves. The small peak of Cr-quinate was eluted from 1.9 to 2.5 min, Cr-malate peaks were eluted from 2.5 to 3.3 min, 3.3–4.9 min, and 4.9–5.7 min, and Cr-aconitate peak, the most intensive in leaf sap, was eluted from 5.7 to 6.9 min.

The quantification of separated Cr species by post column ID-ICP-MS in plant sap further revealed that after the exposure of the dandelion to Cr(VI), the sum of Cr species was slightly lower in root sap than in the root leaves (33.1 and 35.5 mg kg^−1^ Cr, respectively). After reducing the Cr(VI) in the dandelion root, the dominant peak (17.2 mg kg^−1^ Cr) associated with Cr-aconitate accounted for 52% of Cr species eluted from the column. During the translocation of Cr from root to leaf, a significant amount of Cr-aconitate was transformed to Cr-malate. In dandelion leaf sap, Cr-malate with a concentration of 29.3 mg kg^−1^ Cr denoted 83% of the Cr species present in the leaf sap. The data in Table 2 also show that the sum of the concentrations of Cr species eluted from the column, compared to total Cr content, represents about 70% of the Cr species in the dandelion root or leaf sap.

The uptake of Cr in dandelion exposed to Cr-nitrate (pH 6.5) was significantly lower than after exposure to Cr(VI). This is in line with the findings of Huang et al. (2018), who reported that negatively charged oxyanion CrO_4_^2-^ passes the plasma membrane through the sulfate/phosphate carrier system much more easily than Cr-nitrate, which is mostly regulated by a passive transport mechanism and is present at pH 6.5 as a neutral species Cr(OH)_3_^0^. It can be further seen that after the exposure to Cr-nitrate, Cr was mostly accumulated in the root. The sum of the Cr species in the root sap was 16.1 mg kg^−1^ Cr, while in the leaf sap it was 7.16 mg kg^−1^ Cr. Cr-aconitate was the major Cr(III) complex found in the root sap (8.38 mg kg^−1^ Cr) and the leaf sap (4.36 mg kg^−1^ Cr), representing 52 and 60% of the Cr species eluted from the column, respectively. From the data in Table 2, the sum of the concentrations of Cr species eluted from the column, compared to total Cr content in the root and leaf sap, represents about 60 and 75%, respectively.

The main findings from the experimental results of the speciation analysis in which the HPLC-ICP-MS and HR-MS measurements were carried out were as follows. In the dandelion root and leaf sap in the plant grown in the soil with the high Cr content at Vranja Peč, Cr was found to be present as Cr-aconitate, Cr-malate and Cr-quinate complexes, while the Cr-quinate complex was not detected in the leaf sap. In dandelion that was exposed to solutions with a high concentration of Cr(VI), the Cr(VI) was completely reduced in the dandelion root and metabolized into complexes of Cr-aconitate, Cr-malate, and Cr-quinate, and transferred in the same species to the leaf. Dandelion that was exposed to a solution of Cr-nitrate (pH 6.5) was also metabolized in the root to Cr-aconitate, Cr-malate and Cr-quinate complexes, which were translocated to the leaf.

Knowledge of the Cr metabolism in plants significantly contributes to the development of methods for the effective bioremediation of Cr with plants. As expected, our study confirmed that Cr(VI) cannot exist in plants as it is completely reduced to Cr(III) and complexed with LMM organic ligands. The possibility of identifying and quantifying Cr(III) complexes provides new insights into the occurrence and transformation of Cr species in plants.

### 3.4 Localization of Chromium in Dandelion Leaf by LA-ICP-MS

To localize the Cr in the dandelion leaf, bio-imaging was performed with LA-ICP-MS using the procedure described in Section 2.7.

Because of the large area of the dandelion’s leaf, the measurement parameters for the LA-ICP-MS imaging were adjusted to achieve good resolution, while maintaining a reasonable analysis time and adequate sensitivity. For this purpose, a spatial resolution of 40 × 35 µm per pixel was achieved with the laser beam parameters set to a square spot of 35 × 35 µm and a scanning speed adjusted to 200 µm s^−1^. Due to the uneven thickness of the leaf sample, the laser fluence was set to 3.5 J cm^−2^ with a 50 Hz firing rate to achieve total ablation of the sample. The dwell time per cycle was set to 200 ms. However, due to the thickness of the leaf veins and the laser focus, some parts of the veins were not completely ablated (mostly a small amount of primary leaf vein remained).

In order to determine the spatial distribution and the amount of Cr in the dandelion leaf, 0.45 µm nitrocellulose filters with an added Cr standard solution were used as a calibration standard. These filters were chosen because of their similar composition to the plant matrix, easy and rapid preparation, surface smoothness (even and total ablation), known thickness, ability to absorb the Cr standard solution, and (by cutting the central part out) avoiding the “coffee stain” effect caused by drying of the filter. The total Cr concentration of the digested filter calibration standards (average of six replicates) measured with the ICP-MS is shown in Supplementary Table S3. In the filter standards, the even Cr distribution was confirmed with LA-ICP-MS, by ablation and the measurement of Cr in parallel lines running across the entire length of the filter cutouts. During the Cr mapping of the dandelion leaf the calibration standards were measured multiple times. After 50 ablation lines of the dandelion sample, each standard was ablated once with the same instrument parameters. Knowing the concentration and mass of the ablated calibration material per pixel, we were able to determine the total Cr mass in the pixel of the leaf sample from the signal intensity. However, due to the uneven thickness, and lack of thickness information for each part of the leaf sample, this quantification approach is only semi-quantitative and the Cr concentration is given as total Cr amount in the pixel area (40 × 35 µm).

After the filter ablation, we observed that a small quantity of filter particles was still crumbling from the ablation crater about a minute after the ablation, which contributed to the slightly higher concentration lines, which can be seen on the Cr map in the leaf image.

**FIGURE 6:**
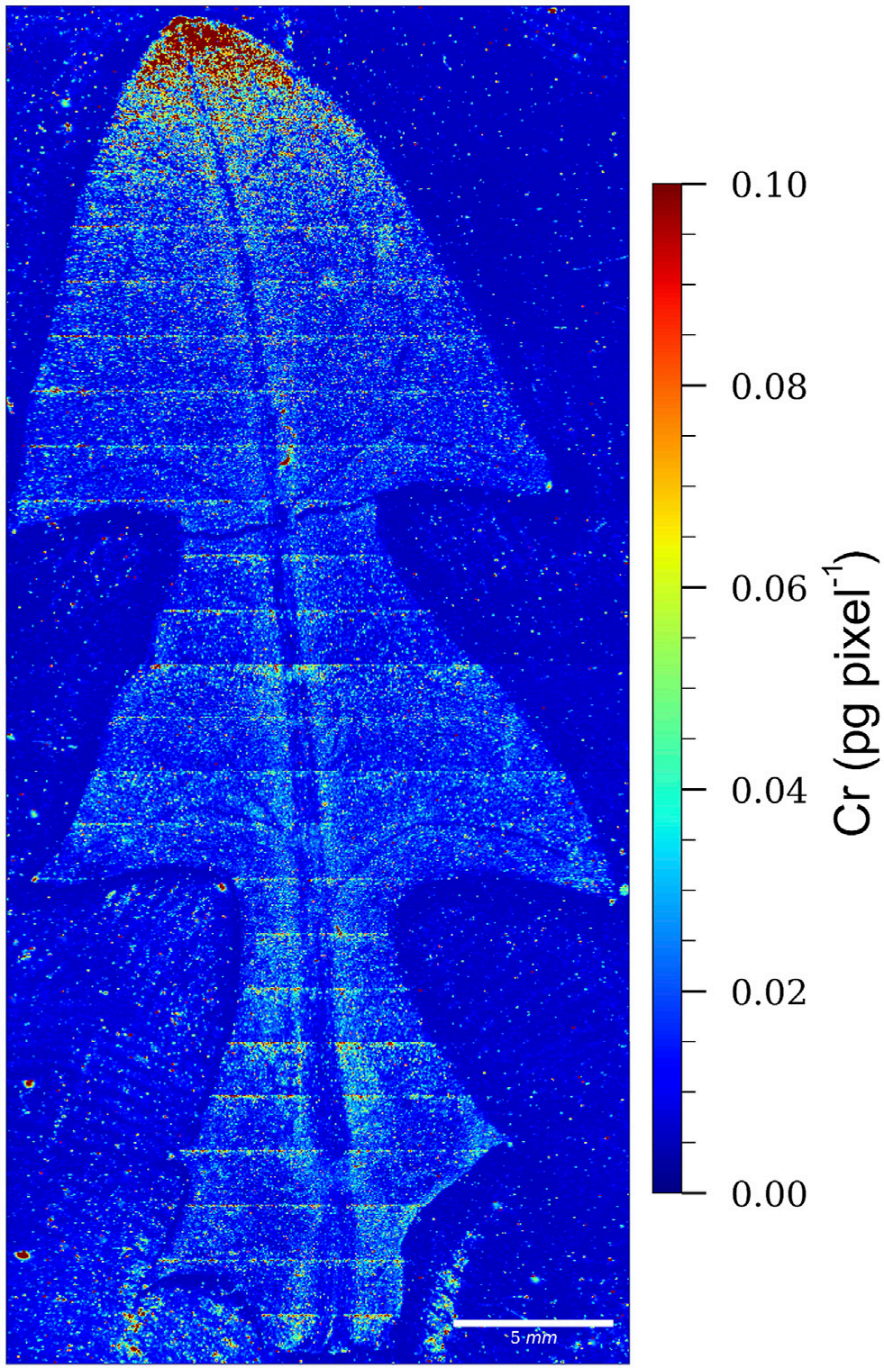
LA-ICP-MS image of a dandelion leaf from Vranja Peč, showing the relative distribution of Cr. The color scale (red to blue; highest to lowest) denotes the Cr concentrations.

The LA-ICP-MS image of a dandelion leaf from Vranja Peč showing the relative distribution of Cr is presented in Figure 6.

From the relative distribution of Cr and the intensity of the red color, which indicates the highest Cr pixel concentration, it is evident that Cr in the leaf of the dandelion growing in the soil from Vranja Peč is localized mainly at the apex of the leaf. This means that Cr species are relatively mobile during the transport through the leaf.

## 4 Conclusions

The original analytical procedure for the speciation of individual Cr species in the dandelion plant (*Taraxacum officinale*) was developed using anion-exchange HPLC-ID-ICP-MS for species separation and HR-MS for ligand identification. In synthetic solutions, Cr-oxalate, Cr-malate, Cr-citrate, Cr-aconitate, and Cr-quinate complexes, as well as Cr-nitrate (pH 6.5) were separated from Cr(VI). To the best of our knowledge, this is the first report that allows speciation of the above mentioned individual Cr(III) complexes and Cr(VI). Using the developed analytical procedure, Cr speciation was performed in the roots and leaves of dandelions grown in Cr-rich soil. The uptake and metabolism of the Cr(VI) and Cr-nitrate (both at pH 6.5) in the dandelions were also investigated. The results showed that for dandelions grown in Cr-rich soil, and dandelions exposed to Cr-nitrate solution, the Cr was accumulated mostly in the roots, while in the dandelions exposed to Cr(VI) solution, the Cr was evenly distributed between the roots and the leaves. The predominant Cr species found in the dandelion plant were Cr-aconitate and Cr-malate, with traces of Cr-quinate. Data from the present study confirmed that the Cr(VI) was detoxified in the dandelion roots by its complete reduction to the far less toxic Cr(III) complexes. The LA-ICP-MS data, which provide supplementary information to the speciation analysis data, showed that the Cr in the leaf of the dandelions grown in Cr-rich soil was localized mainly at the apex of the leaf. The developed analytical methodology can also be used for Cr speciation in other plant samples. Our research makes an important contribution to understanding the transport, metabolism and detoxification processes of Cr in plants and the knowledge that can be used in planning effective remediation strategies using plants.

## Data Availability

The raw data supporting the conclusions of this article will be made available by the authors, without undue reservation.
