# Supplementary material for: Speciation and Bio-Imaging of Chromium in Taraxacum officinale Using HPLC Post-column ID-ICP-MS, High Resolution MS and Laser Ablation ICP-MS Techniques
Source: Front Chem. 2022 May 26;10:863387. doi: 10.3389/fchem.2022.863387 (PMC9177994; doi:10.3389/fchem.2022.863387)
Supplement: Supplementary file 1 [file Table1.DOCX]

Supplementary Material

**Supplementary Table 1.** Instrumental operating parameters.

**ICP-MS operating parameters**

| Method | HPLC-ICP-MS | ICP-MS | LA-ICP-MS |
| --- | --- | --- | --- |
| ICP-MS instrument | Agilent 7900 | Agilent 7900 | Agilent 8800 |
| Parameter | Type/Value  Cr speciation analysis | Type/Value  Cr analysis | Type/Value  Cr analysis |
| *Aerosol introduction* | Miramist | Miramist | ARIS |
| Spray chamber | Scott | Scott | / |
| Skimmer and sampler | Ni | Ni | Ni |
| *Plasma conditions* |  |  |  |
| Forward power | 1550 W | 1550 W | 1550 W |
| Plasma gas flow (Ar) | 15.0 L min^-1^ | 15.0 L min^-1^ | 15.0 L min^-1^ |
| Carrier gas flow (He) | / | / | 0.6 L min^-1^ |
| Carrier gas flow (Ar) | 0.75 L min^-1^ | 1.10 L min^-1^ | 1.05 L min^-1^ |
| Dilution gas flow (Ar) | 0.45 L min^-1^ | 0.15 L min^-1^ | / |
| He gas flow | 10 mL min^-1^ | 4.5 mL min^-1^ | / |
| Total carrier gas flow | 1.20 L min^-1^ | 1.25 L min^-1^ | 1.65 L min^-1^ |
| QP bias | -97 V | -15 V | -2.0 |
| Oct bias | -100 V | -100.0 V | -18.0 V |
| Cell entrance | -115 V | -130 V | -40 V |
| Cell exit | -150 V | -150 V | -50 V |
| Deflect | -74V | -75.0 V | -5.0 V |
| Plate bias | -150 V | -150 V | -50 V |
| Sample-uptake rate | 1.5 mL min^-1^ | 0.3 mL min^-1^ | / |
| *Data-acquisition parameters* |  |  |  |
| *m/z* of isotopes monitored | ^50^Cr, ^52^Cr | ^52^Cr | ^52^Cr |
| *m/z* of internal standard | ^103^Rh | ^103^Rh | ^/^ |
| Total acquisition time | 600 s | ^/^ | / |

**Laser operating parameters**

| Instrument | Analyte G2 | |
| --- | --- | --- |
| Parameter | Type/Value | |
| Wavelength | 193 nm |  |
| Sample chamber | HelEx-II | |
| Laser energy (Fluence) | 3.5 J cm^-2^ | |
| Laser- beam size | 35 µm × 35 µm (square) | |
| Scanning speed | 200 µm s^-1^ | |
| Repetition rate | 50 Hz | |
| Carrier gas flow (He) | 0.60 L min^-1^ | |

**ESI-MS operating parameters**

| ESI-MS Instrument | Q-TOF Premier (Waters) |
| --- | --- |
| Parameter | Type/Value  MS analysis |
| Electrospray ionisation | Negative mode (ESI-) |
| Capillary voltage  Sampling zone  Nebulizer gas  Mobile phase  Source temperature  Desolvation temperature  Mass resolution  Lock spray interface mass  Lock mass solution  Collision gas  Collision voltage | 2,5 kV  20 V  Nitrogen  Water-Acetonitrile1:1  100 ^0^C  300 ^0^C  10000  554,2615  Leucine-Enkephalin  Argon  20 V |
| Mass range | m/z 50-1000 |
| Scan time | 1 s |
| Total acquisition time | 240 s |

**Supplementary Table 2.** Concentrations of Cr in standard reference material SPS-SW1 (Reference material for measurements of elements in surface waters) determined by ICP-MS, and in certified reference materials CRM 320R (Trace Elements in River Sediment) and SRM 1573a Tomato Leaves determined by ICP-MS after microwave-assisted digestion. The results represent the mean concentration from three parallel samples.

| Reference material | Certified Cr concentration | Determined Cr concentration |
| --- | --- | --- |
| SPS-SW1 (ng mL^-1^) | 2.00 ± 0.02 | 1.97 ± 0.03 |
| CRM 320R (mg kg^-1^ d.w.) | 59 ± 4 | 56 ± 2 |
| SRM 1573a (mg kg^-1^ d.w.) | 1.988 ± 0.034 | 1.95 ± 0.06 |

**Supplementary Table 3.** Total Cr concentration of the digested filter calibration standards (average of six replicates with corresponding RSD) measured by ICP-MS.

| Sample | Cr concentration (mg kg^-1^) | RSD (%) |
| --- | --- | --- |
| Standard 1 | 0.904 | 15.6 |
| Standard 2 | 1.70 | 4.7 |
| Standard 3 | 6.0 | 11.6 |
| Standard 4  Standard 5 | 16.3  60.1 | 7.8  3.9 |

| **** |
| --- |

**Supplementary Figure 1.** Speciation of Cr in synthetic solutions of Cr-nitrate, containing 50 ng mL^-1^ Cr, prepared in 0.013 M phosphate buffer (pH 6.5) applying the HPLC-ICP-MS procedure.

|  |  |
| --- | --- |

**Supplementary Figure 2.** Speciation of synthetic solutions of Cr-nitrate and Cr-malate (50 ng mL^-1^ Cr) at pH 3.5 and pH 6.5 applying HPLC-ICP-MS procedure.

| A |  |
| --- | --- |
| B |  |

**Supplementary Figure 3.** High-resolution ESI-Q-TOF mass spectrum of **(A)** dandelion root sap and **(B)** dandelion leaf sap in mass range *m/z* 50 to *m/z* 200.

| Dandelion root | | |
| --- | --- | --- |
|  |  |  |
| **** | **** | **** |
| Cr-quinate (1.9–2.5 min) | Cr-malate (2.5–3.0 min) | Cr-malate (3.0–4.9 min) |
|  |  |  |
| **** | **** |  |
| Cr-malate (4.9–5.5 min) | Cr-aconitate (5.5–7.0 min) |  |
|  | | |
| Dandelion leaf | | |
|  |  |  |
|  |  | **** |
| Cr-quinate (1.9–2.5 min) | Cr-malate (2.5–3.0 min) | Cr-malate (5.0–5.8 min) |
|  |  |  |
|  |  |  |
| Cr-aconitate (5.8–6.3 min) |  |  |

**Supplementary Figure 4.** HR-MS spectra of the Cr species eluted under the chromatographic peaks of dandelion root and leaf sap, exposed for 48 h to synthetic solutions of Cr(VI).

| Dandelion root | | |
| --- | --- | --- |
|  |  |  |
| **** | **** | **** |
| Cr-malate (0.7–1.2 min) | Cr-quinate (1.9–2.5 min) | Cr-malate (2.5–3.3 min) |
|  |  |  |
| **** | **** | **** |
| Cr-malate (3.3–4.9 min) | Cr-malate (4.9–5.7 min) | Cr-aconitate (5.7–7.2 min) |
|  | | |
| Dandelion leaf | | |
|  |  |  |
|  |  | **** |
| Cr-quinate (1.9–2.5 min) | Cr-malate (2.5–3.3 min) | Cr-malate (3.3–4.9 min) |
|  |  |  |
|  |  |  |
| Cr-malate (4.9–5.7 min) | Cr-aconitate (5.7–6.9 min) |  |

**Supplementary Figure 5.** HR-MS spectra of the Cr species eluted under the chromatographic peaks of dandelion root and leaf sap, exposed for 48 h to synthetic solutions of Cr-nitrate.
